# The Influence of the Consumer Ethnocentrism and Cultural Familiarity on Brand Preference: Evidence of Event-Related Potential (ERP)

**DOI:** 10.3389/fnhum.2019.00220

**Published:** 2019-07-04

**Authors:** Qingguo Ma, H’meidatt Mohamed Abdeljelil, Linfeng Hu

**Affiliations:** ^1^School of Management, Zhejiang University, Hangzhou, China; ^2^Institute of Neural Management Sciences, Zhejiang University of Technology, Hangzhou, China; ^3^Academy of Neuroeconomics and Neuromanagement, Ningbo University, Ningbo, China; ^4^School of Management, Zhejiang University of Technology, Hangzhou, China

**Keywords:** event-related potential, N200, consumer ethnocentrism, preference, neuromarketing, neuromanagement, branding, purchase intention

## Abstract

The tendency of customers’ preference to their local brands over the foreign ones is known as consumer ethnocentrism, and it is an important issue in international marketing. This study aims at identifying the behavioral and neural correlates of Consumer Ethnocentrism in the field of brand preference, using event-related potential (ERP). We sampled subjects from two ethnic groups, a Chinese ethnic group and a sub-Saharan Black African group from Zhejiang University. The subjects faced two sequential stimuli, S1 followed by S2. S1 consisted of 40 pictures of 20 Chinese and 20 Black Africans people wearing traditional clothes, and S2 consisted of 40 fake brand-logos which were divided randomly into two groups of 20 each. The subjects were informed that the people in S1 purchased and recommended the products with the brand-logos presented in S2, and the subjects were asked to rate their preference degree toward these logos. The brand-logos were called the “in-group recommended logos” if the recommenders in S1 were the same race as the subjects, otherwise, the “out-group recommended logos.” The results revealed that the race of the brand-logo recommender highly impacted the Chinese subjects’ preference for the brand-logos. The N200 component elicited by the in-group recommended logos were significantly lower than those elicited by the out-group recommended logos. Additionally, there was evidence that being familiar with foreign cultures reduced consumer ethnocentrism. The African subjects were familiar with Chinese people and adopted a Chinese culture, as a result, they did not differ in showing preferences between the in-group and out-group recommended logos.

## Introduction

Consumer’s tendency to buy a locally made good over a foreign product is known as consumer ethnocentrism (de Ruyter et al., 1998; Moon and Jain, 2002; Rand et al., 2009; Jiménez and San Martín, 2010). This conceptual phenomenon leads to making purchasing decisions that do not only depend on price-quality but also depend on the criterion of where the product comes from (Garmatjuk and Parts, 2015). Ethnocentrism is originally a sociological concept, and it shows the relationship between a group to which an individual belongs (in-group) and the group that the individual does not belong to (out-group; de Ruyter et al., 1998). A group is not limited to a social or racial segregation but could also be any other organization that an individual feels apart of, such as gender, ethnicity, religion, musical preferences, dressing styles (Hornstein, 1972; Fershtman and Gneezy, 2001; Platow and van Knippenberg, 2001; Levine et al., 2005; Stürmer et al., 2006; He et al., 2009; Rand et al., 2009), and it is a process which starts from childhood to adulthood (Ben-Ner et al., 2009). Individuals from a cultural collectivism like the Chinese and Africans often see their role as individuals in relation to the family or society that they identify themselves with and, therefore, support in-group members (Chen et al., 2002; Hustinx and Lammertyn, 2003; Kemmelmeier et al., 2006; Pierre and Matondo, 2012).

In the current study, the priming stimuli consisted of two ethnic group pictures, Black African and Asian Chinese people. Generally, during race facial differentiation the subjects have some difficulty in identifying the other races’ face, which is known as other race effects (Sporer, 2001; Hugenberg et al., 2007). Some previous research that studied faces of an in-group and out-group race concluded that three event-related potential (ERP) components could be distinguished between in-group and out-group race faces, comprising the early negative component which reflects the face processing and peaking around 170 ms after the stimulus (N170), the negative wave recorded between 200 and 350 ms after the stimulus (N200), and the late positives potential appearing around 300 ms after the stimulus (P300; Dickter and Bartholow, 2007; Walker et al., 2008). Furthermore, the ERP components P200 and the N200 were linked with early attention processes while the P300 was associated with the evaluative categorization processes (Ito et al., 1998; Dickter and Bartholow, 2007; Walker et al., 2008). During the categorization of the in-group and out-group faces, the existing results showed that the own race faces elicited larger N200 at the frontal area while the other race faces elicited larger P200 and P300 at the parietal region (Dickter and Bartholow, 2007). Another experiment which used an in-group face, an out-group face and a non-face as stimuli found that N170 distinguished the face stimuli from the non-face stimuli, and the N250 elicited by the in-group faces was larger than that of the out-group faces (Ito et al., 2004). In addition, the same study revealed that only in the people with higher levels of prejudice the late-positive potential (LPP) peaking at partial electrode differentiated between the in-group and out-group faces (Ito et al., 2004). However, the familiarity and contact with the other culture-ethnic group could reduce all racial bias aspects (Malinowska, 2016). Previous researchers, for example, found that cultural familiarity and contact with other ethnicities reduced the racial bias in empathy (Xu et al., 2009; Zuo and Han, 2013). Moreover, after the training which aimed at increasing the level of familiarity with the other group race face, the amplitude of N250 for the other race group face was larger than before (Tanaka and Pierce, 2009). Meanwhile, the existing studies found that N170 could not differentiate between familiar and unfamiliar faces (Jemel et al., 2003; Tanaka et al., 2006). However, some previous studies suggested that the N250 elicited by familiar faces was connected solely to one semantic process which was related to familiar face identification (Bentin and Deouell, 2000), while during the interaction of familiarity and facial expression processing the familiar faces elicited a shorter P300 latency than the unfamiliar faces (Wild-Wall et al., 2008).

Related studies using neuroscience to investigate consumer ethnocentrism are scarce. Until now, most scholars have used a consumer ethnocentrism tendencies scale approach, which is composed of 17 items, to study consumer ethnocentrism (Shimp and Sharma, 1987). Although the concept of ethnocentrism refers to the preference of in-group over out-group products regardless of the group category (nation, ethnicity, etc.), in practice the majority of consumer ethnocentrism research focuses mainly on the nation-state group. For example, in different countries, consumers driven by patriotism tend to prefer local products over imported ones (Shimp and Sharma, 1987; Vida et al., 2008; Wise, 2017). Only a few studies used the category group, other than a nation-state, such as Vida et al. (2008) who distinguished between the in-group and out-group based on the culture-ethnic groupings. Moreover, consumer ethnocentrism could be driven by other factors than patriotism. These other factors could include perceived vulnerability to a threat (Wise, 2017), brand and product category (Balabanis and Siamagka, 2017) and cultural identity (He and Lu, 2015). The theory of planned behavior (TPB) is frequently used to predict the purchase intention and brand preference, which claims that attitude, subjective norms and the ability to do any specific behavior, determines the behavior intention (Ajzen, 1991). Typically, subjective norms are divided into two parts, the descriptive norm, and the conjunctive norm. Descriptive norm refers to an individual doing what the others do, i.e., the individual adopts the others’ opinions and behaviors (Stok et al., 2014). Conjunctive norms are defined as what an individual should do, is determined by acceptable and unacceptable social group behavior (Stok et al., 2014). Although the original TPB did not distinguish between the two types of norms, the descriptive norm was nonetheless often used to predict the behavioral intentions (Borsari and Carey, 2003). The term social conformity was used widely by neuroscience scholars to describe the effect of the group opinion on individual behavior (Stallen and Sanfey, 2015). However, EEG studies found that the conformity related to the brain activities correlated to the two brain areas, the striatum and the ventromedial prefrontal cortex (Bartra et al., 2013; Stallen and Sanfey, 2015). On the other hand, several studies have linked EEG frontal asymmetries to consumer choice prediction and purchase intention (Ambler et al., 2004; Vecchiato et al., 2011). Besides, ERP components were associated with categorizing and evaluating stimuli, P300 (Polich, 2007) and N200 (Folstein and Van Petten, 2008) and most recently P200 and LPP (Ma et al., 2018). Furthermore, ERP component N200 and a weaker theta band was associated to purchase intention (Telpaz et al., 2015).

Cultural familiarity is a large topic which entails several dimensions (e.g., experience; Carneiro and Crompton, 2010). Commonly, the number of previous visits to a destination was used as criteria to distinguish between the familiar and unfamiliar groups (Carneiro and Crompton, 2010; Prats et al., 2016). A tourist’s previous visits and experiences influence the positivity of their satisfaction about the destination (Prats et al., 2016; Trianasari et al., 2018). Moreover, a tourist’s familiarity has a positive impact on the image of the local products of the destination and induces a higher intention for consuming local products such as food (Seo et al., 2013). Furthermore, cultural sensitivity also positively and directly impacts the image of the destination and the visitors’ satisfaction (Palacio and Martín-Santana, 2018). The cultural sensitivity measures the cultural openness, which refers to the willingness to communicate with people from different culture-ethnic groups and to experience their related objects (Sharma et al., 1995; Shankarmahesh, 2006; Mahon and Cushner, 2014). To the best of our knowledge, none of the consumer ethnocentrism scholars have explored the relationship between consumer ethnocentrism and cultural familiarity, while several studies have investigated the relationship between cultural sensitivity and consumer ethnocentrism (Sharma et al., 1995; Nguyen et al., 2008; Wang, 2018). Consumers with a high degree of cultural sensitivity are more positive and feel less threatened by the other culture-ethnic groups and consequently, such consumers prefer more of the imported products than the consumers with a lower degree of cultural sensitivity (Wang, 2018).

In this experiment, we sampled subjects from two groups consisting of Asian Chinese people who had never been outside of China and Black people of sub-Saharan African origin who have been international students in China for more than 1 year, respectively. In order to understand the effect of the cultural familiarity on consumer ethnocentrism based on ethnic-culture groupings and driven by descriptive norms, we applied an ERP experiment with the S1–S2 paradigm. S1 presented pictures of the two races with neutral facial expressions and wearing ethnically corresponding traditional clothes. The two race groups’ pictures were then followed by S2, fake brand logos (S2) which were randomly divided into two groups, one corresponding to Chinese people’s pictures (referred to as C-logo), and the others corresponded to the African people’s pictures (A-logo). The subjects were informed that the people in S1 had bought the earphones and therefore recommended the brand-logo in S2 to them. The subjects were then asked to indicate their preferences for the logos. The logos were called the “in-group recommended logos” if the recommenders in S1 were the same race as the subjects, otherwise, the “out-group recommended logos.”

Based on the above introduction, we hypothesized that cultural familiarity would modulate consumer ethnocentrism driven by descriptive norms, i.e., the impact of the recommendation of the race picture in S1 on the brand-logo in S2 was determined by the cultural familiarity of Chinese and African subjects. In Chinese subjects, the unfamiliarity to the African culture and people was expected to lead them to be more bias toward the Chinese than the African recommender in S1, which made the Chinese subjects prefer the C-logo more than the A-logo. In the African subjects, the familiarity with the Chinese culture and people reduced the racial bias in S1, thus no difference in preference was expected between the A-logo and C-logo.

## Materials and Methods

### Participants

Twenty international male students who have lived in China for more than 1 year and identify themselves as being of Black ethnicity with sub-Saharan origin were engaged in the group of Black African subjects (age range = 19–37 years, M = 25.95, SD = 6.41), and 20 Asian Chinese males (age range = 20–32 years, M = 26.3, SD = 3.12) who identify themselves as being of Asian ethnicity and who have not traveled outside of China were enlisted to denote the Asian Chinese subjects in this study. The independent sample *t*-test revealed no significant difference of age between the two subject groups, *t*_(38)_ = −0.157; *p* = 0.876. Both Chinese and African subjects were students at Zhejiang University. The experiment was conducted at the Neuro-management lab, Zhejiang University. All subjects had normal or corrected to normal vision with no history of neurological or psychiatric abnormalities. This study was approved by the Neuromanagement Laboratory Ethics Committee at Zhejiang University. Written informed consent was obtained from all participants before the ERP experiment.

### Stimuli

In the current study, the priming stimuli (S1) consisted of 40 pictures of 20 Asian Chinese and 20 Black Africans (equal number of males and females) with neutral facial expressions and wearing corresponding traditional clothes, which were chosen randomly from the Internet. All the people in the pictures stood up, simply extended their hands without a hand gesture, and had the same posture. The subjects were not familiar with race pictures and did not include celebrity pictures. These pictures were processed to have the same size and background using Adobe Photoshop 13.0.S2, consisted of 40 fake earphone logo pictures used in our previous study (Ma et al., 2017) and were divided randomly into two groups of 20 each, following Chinese pictures and African pictures, respectively, in the ERP experiment.

To test the stimuli, all subjects were asked to rate for the attractiveness of the race stimuli (S1) and preference of the logo stimuli (S2) on a 5-point scale (1 = very low to 5 = very high), after the ERP experiment section. The differences between the Chinese Race Picture and African Race Picture in attractiveness were rated by each subject group. And a significant difference was found in the Chinese subject group (M_Chinese-Picture_ = 3.785, SD_Chinese-Picture_ = 0.446; M_African-Picture_ = 2.23, SD_African-Picture_ = 0.345; *t*_(19)_ = 17.162, *p* = 0.000), while in African subject group there was no significant difference (M_Chinese-Picture_ = 3.210, SD_Chinese-Picture_ = 0.65; M_African-Picture_ = 3.265, SD_African-Picture_ = 0.589; *t*_(19)_ = −0.704, *P* = 0.49). In addition, The two groups of logos, i.e., C-logos and A-logos, were tested without priming stimuli in Chinese subjects in the previous ERP study, and no difference between the two logo groups was found (Ma et al., 2017). Thus we just asked the African subjects to rate the two groups of logos without priming stimuli, the paired sample *t*-test revealed that there was no difference in the preferences between the A-logos and C-logos (M = 2.798, SD = 0.33) and C-logos (M = 2.93, SD = 0.304), *t*_(19)_ = −1.146, *p* = 0.266.

The subjects’ familiarity was measured by their previous visits to a destination according to previous studies (Carneiro and Crompton, 2010; Prats et al., 2016). Specifically, the Chinese subjects were asked; “Have you ever been to Africa?” The question required a binary response of Yes/No. If the answer was yes, then the follow-up question was “How long have you stayed in Africa?” The African subjects were asked the same questions about their visit to China. The result showed that all the Chinese subjects had never been to Africa, while all the African subjects were international students living in China for 16 months on average. The subjects were also given a cultural sensitivity scale (Loo and Shiomi, 1999; Nguyen et al., 2008) for supplementary analysis. The scale consists of five items Likert scaled (from 1 = strongly disagree to 5 = strongly agree). Cronbach’s alpha of the five items was 0.74. The independent sample *t*-test of the culture sensitivity scale revealed that the African subjects had a higher mean score (3.8 ± 0.349) compared to the Chinese subjects (2.7 ± 0.358); *t*_(38)_ = −9.828, *p* = 0.000.

### Experimental Procedures and EEG Recording

**Figure 1:**
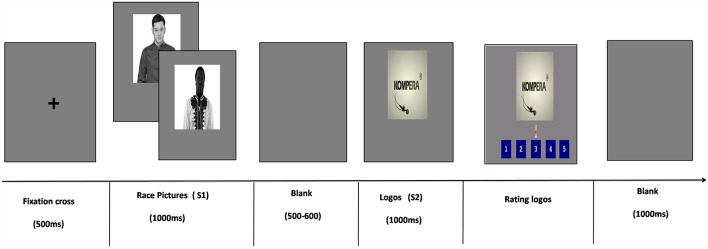
The event-related potential (ERP) experiment procedure. Participants saw a Chinese Asian picture, or an African Black picture at first (S1), then a logo (S2). The subject is informed that the people in S1 purchased and positively recommended the logo in S2 to them. Their task was to rate logos using the mini keypad.

Figure 1 indicates a single trial in the ERP experiment. The stimuli were presented using the E-prime 2.0 software package (Psychology Software Tools, Pittsburgh, PA, USA). A fixation appeared at the beginning of each trial for 500 ms on a gray screen. Next, a picture of Chinese or African people (S1) was presented for 1,000 ms. Then a gray blank screen was presented randomly between 500 ms and 600 ms, and then, a picture of the logo (S2) was subsequently presented for 1,000 ms. The subjects were told that people appearing in the S1 pictures currently use and therefore recommend the earphones in S2 to them, and their task was to rank the logo in S2 from 1 (dislike a lot) to 5 (like a lot) using the mini keypad. After the rating, a 1,000 ms blank was presented at the end of each trial.

In total 80 trials were randomly presented to each subject in this experiment, i.e., 40 Race Pictures in S1 (20 Asian Chinese pictures, 20 Black African pictures) and then 40 logos in S2 (20 C-logos and 20 A-logos), presented to each subject twice.

EEGs were recorded (band-pass 0.05–70 Hz, sampling rate 500 Hz) with a NeuroScan SynAmps2 Amplifier (Scan 4.3.1, Neurosoft Labs, Inc., Sterling, VA, USA), using a 64-channel electro-cap with Ag/AgCl electrodes, in mounted according to the extended International 10-20 system and referenced to linked mastoids. Vertical and horizontal electrooculograms were recorded with two pairs of electrodes, one placed above and below the right eye, and another 10 mm from the lateral canthi. The electrode impedance was maintained below 5 kΩ during the experiment.

### Data Analysis

EEG data were analyzed using the software NeuroScan version 4.3.1. The EOG artifacts were initially corrected, followed by digital filtering through a zero-phase shift (low pass at 30 Hz, 24 dB/octave). The EEGs were segmented for 1,000 ms in each epoch, 200 ms before the onset of S1 and S2 until 800 ms after the onset, respectively. The baseline corrected using the 200 ms before the stimulus onset. Trials that contained amplifier clippings, bursts of electromyography activity, or peak-to-peak deflection that exceeded ±80 μV were excluded from the final average.

Based on visual inspection, the time window of P300 was chosen as 290–420 ms after the onset of the race picture (S1). Besides, P300 after the onset of S1 was observed over the parietal-occipital site that was similar to prior studies (Ito et al., 2004; Dickter and Bartholow, 2007). Thus, we selected the PO5, POZ, and PO6 for our analyses of P300. After the onset of the logo stimulus (S2), the time window of 200–350 ms was chosen for N2. Considering that previous studies of product preference N2 components are generally distributed largely in the frontal and midfrontal regions (Telpaz et al., 2015), we therefore, chose the electrodes F3, FZ, F4, FC3, FCZ, and FC4 for the analyses of N2.

To analyze the neural response of stimuli in S1, we conducted a mixed-model ANOVA with Subject Groups (Chinese subjects vs. African subjects) as a between-subject factor and Race Picture groups (Asian Chinese people vs. Black African people) as a within-subject factor on P300. Whereas, to analyze the impact effect of S1 on brand-logos stimuli in S2, we performed a mixed ANOVA with the Subject Group as the between-subjects factor and Logo groups (C-logos vs. A-logos) as the within-subject factor on the N2 component. The brand-logos rating score was analyzed using a mixed-model ANOVA with the Subject Group (Chinese subjects vs. African subjects) as the between-subject factor and brand-logos Groups (C-logos vs. A-logos) as a within-subject factor. After the mixed ANOVA for both behavior and neural data, the paired sample *t*-tests were used to break down the interaction effects revealed in those ANOVA analyses (Raz et al., 2013). All the data related to this study are provided in Supplementary Table S1. The Greenhouse-Geisser and Bonferroni corrections were applied where appropriate.

## Results

**Figure 2:**
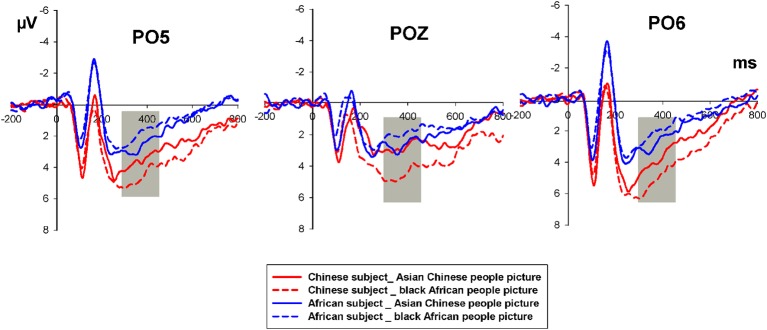
Grand-averaged ERPs of P300 by Race Picture (S1) in the Chinese and African subject groups, respectively. The solid red line refers to Asian Chinese people pictured in S1 for the Chinese subject and the short-dash red line to Black African people pictured in S1 for Chinese subjects. The solid blue line refers to Asian Chinese people pictured in S1 for African subjects and the short-dash blue line to Black African people pictured in S1 for African subjects.

For Race picture (S1) processing, the mixed-model ANOVA on the P300 component revealed that there were significant main effects for the Subject Groups (*F*_(1,38)_ = 4.714, *P* = 0.036) and not for Race Pictures (*F*_(1,38)_ = 2.911, *P* = 0.096). The interaction between the Race Picture and Subject Group was significant (*F*_(1,38)_ = 10.076, *P* = 0.03). The paired sample analysis showed that the mean amplitude of P300 elicited by the Asian Chinese race pictures in the Chinese subject group (3.6142 μV ± 2.072) was significantly lower than that of the Black African race picture (5.19 μV ± 2.93) *t*_(19)_ = −2.953, *P* = 0.008, whereas no significant difference in P300 elicited by the Asian Chinese race picture with that of the Black African race pictures was found in the African subject group *t*_(19)_ = 1.303, *P* = 0.208, Figure 2.

**Figure 3:**
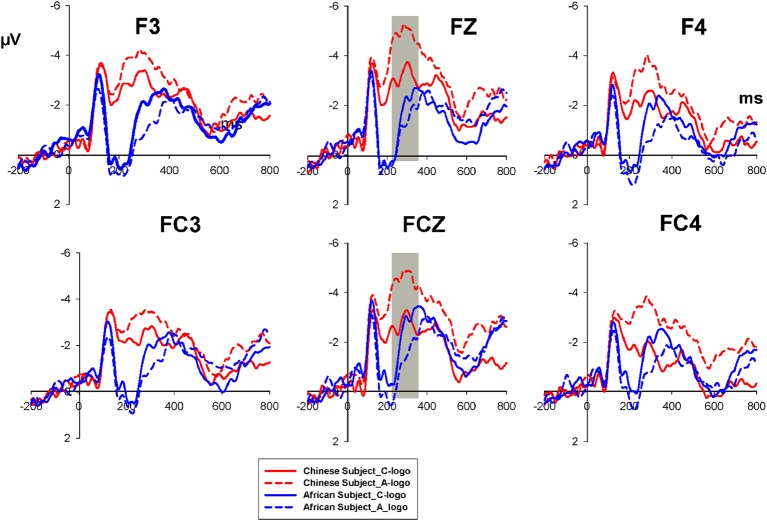
Grand-averaged ERPs of N2 elicited by logo stimuli. The solid red line refers to C-logo for Chinese subjects and the short-dash red line to the A-logo for Chinese subjects. The solid blue line refers to C-logos for African subjects, and the short-dash blue line to the A-logo for African subjects.

According to the results of the mixed ANOVA on N2 for the logo stimuli (S2), as indicated by Figure 3, there was a significant main effect for the Subject Group (*F*_(1,38)_ = 6.917, *P* = 0.012). While the main effect of the logo Picture was not significant (*F*_(1,38)_ = 1.451, *P* = 0.236). In addition, the ANOVA showed a significant interaction effect between the Subject Group and the Logo Pictures (*F*_(1,38)_ = 9.445, *P* = 0.004). The simple effect analysis revealed that in the Chinese subject group the N2 elicited by C-logos was significantly higher (−2.895 μV ± 3.504) than that by A-logos (−4.0928 μV ± 3.372), *t*_(19)_ = 2.854, *P* = 0.01, whereas in the African subject group, no such significant difference was found (*t*_(19)_ = −1.411, *P* = 0.174).

**Figure 4:**
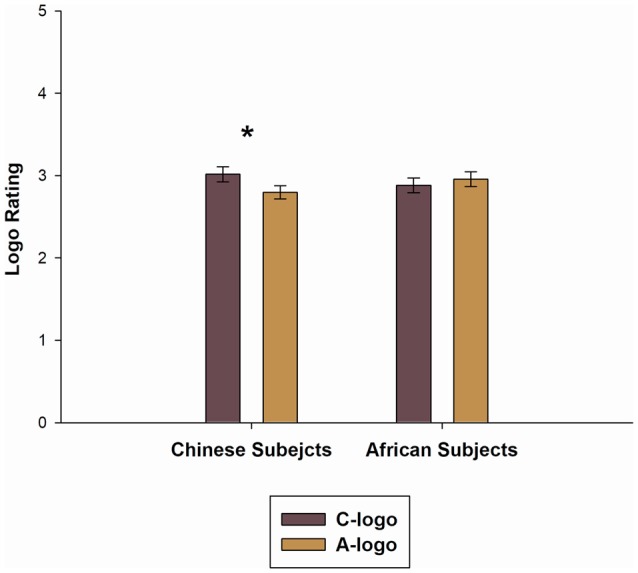
The means of A-logo and C-logo rating scores for the Black African and Asian Chinese subjects. *Denotes a significant difference between the A-logo and C-logo rating for Chinese subjects.

The Mixed-model ANOVA for logo rating (in the ERP experiment) revealed no significant main effects for Subject Groups (*F*_(1,38)_ = 0.013, *P* = 0.909), moreover, there was no significant main effect for Logo Groups (*F*_(1,38)_ = 2.041, *P* = 0.161). While there was a significant interaction effect of the Subject Group with Logo Groups (*F*_(1,38)_ = 8.828, *P* = 0.005). To break down this interaction, a simple effect analysis showed that the C-logo rating scores in the Chinese subject group (3.016 ± 0.407) was significantly higher than the A-logo rating scores (2.798 ± 0.356), *t*_(19)_ = 2.515, *P* = 0.021, whereas in the African subjects group no significant differences were found between A-logo and C-logo rating scores, *t*_(19)_ = −1.592, *P* = 0.128. This result is presented graphically in Figure 4.

## Discussion

The present study sets out to investigate the influence of consumer ethnocentrism on brand-logo preference using the ERP method. We recruited two subject groups, one consisting of Asian Chinese students and the other comprised of Black international students from sub-Saharan African countries living in China. The purchase behavior and the positive recommendation of the logos in S2 from Asian Chinese or Black African peoples in S1 were important determinants of their preferences but only in the Chinese subjects who had low familiarity with African culture, while the effect of the descriptive norm was not found in the African subjects who were familiar with Chinese culture because of their time spent living in China.

The neural response showed that the race picture (S1) evoked a neural bias against the other race group as indicated by P300. The mixed ANOVA on P300 showed the significant interaction between Subject Group and Race Picture (S1), and the simple analysis revealed that the out-group race picture (i.e., the people in S1 were not of the same race as the subjects) elicited a more positive P300 than the in-group race picture in the Chinese subject group, but no such effect was found in the African subject group. Some previous studies have indicated that the P300 was correlated with other race effects (Ito et al., 2004; Dickter and Bartholow, 2007; He et al., 2009; Tanaka and Pierce, 2009). The P300 was associated with the encoding of familiar and unfamiliar faces during the interaction of facial expression and familiarity processing (Wild-Wall et al., 2008). Therefore, the P300 in S1 could appear as a result of the encoding of the familiar and unfamiliar ethnic faces and clothes in the Race picture stimulus. Importantly, as previous literature revealed that only in the higher prejudice group the LPP could be differentiated between the in-group and out-group faces (Ito et al., 2004). Consistent with these findings, we found that the P300 at the similar brain region could be distinguished between the race pictures only in the Chinese subjects. Larger P300 elicited by out-group race pictures indicated that Chinese subjects are less familiar and have greater bias with African race than with their own race, which could also be supported by the self-reported cultural familiarity and sensitivity. However, the P300 results from African subjects did not provide evidence about the racial bias, which might be attributed to the long living time of African subjects in China.

The ERP component N2 elicited by C-logos was significantly smaller than that by A-logos among the Chinese subjects. A previous study found that N2 was associated with purchase intention, and the products with low or no preference elicited a more negative N2 amplitude than the high preferred product (Telpaz et al., 2015). Accordingly, the effect of descriptive norms on the brand preference was evident in Chinese subjects, i.e., the logos purchased and positively recommended by Asian Chinese people were perceived more favorable than the logos purchased and positively recommended by Black African people. However, in the Black African subjects, no significant difference in N2 was found between the A-logo and C-logo, indicating that the African subjects had a similar preference to logos purchased and recommended by Chinese people and African people. The rating scores for the A-logos and C-logos were in line with the N2 result. The influence of the Race Picture on the logo rating was obviously observed, especially for Chinese subjects. Chinese subjects gave a higher rating score for the in-group recommended brand-logo (C-logo) than the out-group recommended brand-logo (A-logo), while the African subjects showed no significant difference between the rating scores of the A-logo and C-logo. These results might be attributed to the African subjects’ familiarity with both Chinese and African culture, which reduced the effect of descriptive norms on brand preference. A previous multicultural study which investigated the Chinese cultural adoption for international students in China concluded that international students adopted the Chinese culture over time (An and Chiang, 2015). According to the cultural familiarity and sensitivity measurements, the African subjects were familiar with Chinese culture as well as with a higher degree of cultural sensitivity, but the Chinese subjects were unfamiliar with African culture and with a lower degree of cultural sensitivity. Although no previous studies investigated the relationship between the cultural familiarity and consumer ethnocentrism directly, several studies found that tourist’s familiarity with the destination can positively affect their local product consumption and their satisfaction about the destination (Seo et al., 2013; Prats et al., 2016; Trianasari et al., 2018). Additionally, a negative relationship between consumer ethnocentrism and cultural sensitivity was found in previous studies, i.e., consumers with a high cultural sensitivity are more positive and feel less threatened by the other culture-ethnic groups, consequently, such consumers prefer imported products more than consumers with a low cultural sensitivity (Wang, 2018). Our study verified the above results by applying the neuroscience method. Specifically, the neural response to the C-logos and A-logos (reflected by N2) of Chinese subjects indicated the existence of consumer ethnocentrism driven by descriptive norms, while the neural reaction of African subjects verified the reduction effect of race-culture familiarity on consumer ethnocentrism.

The results of this study prove that consumer ethnocentrism could exist during the evaluation and selection of brands matched to ethnic groups as well as the origin of the product (local vs. imported), the in-group recommended logos were treated more favorably than the out-group recommended logos in higher ethnocentrism groups (Chinese subject).

One of the limitations of this study is that all the subjects were young male students, future studies, therefore, need to investigate the neural response of the consumer ethnocentrism effect in both male and female subjects of a broader age range. What is more, besides the culture-ethnic grouping basis, future work can investigate consumer ethnocentrism by recruiting subjects from different group categories the individual subjects feel a part of, such as religion (Hornstein, 1972; Fershtman and Gneezy, 2001; Platow and van Knippenberg, 2001; Levine et al., 2005; Stürmer et al., 2006; He et al., 2009; Rand et al., 2009).

This study suggests several implications for locals in addition to international managers and marketers, whereby associating products with a target consumer culture-ethnic group in advertisements, or other marketing activities can benefit from the consumer ethnocentrism effect, leading to higher engagement with their own brands and products.

## Conclusion

The ERP experiment was conducted to explore the impact of cultural familiarity on the ethnic affiliation of consumer ethnocentrism driven by descriptive norms. The experiment consisted of two stimuli, Asian Chinese and Black African recommenders in the S1 who purchased and recommended headphone brand-logos in S2. The ERP component N2 was enhanced by the in-group recommended logo more than the out-group recommended logos in the Asian Chinese subjects. While no significant difference was found between the two groups of brand-logos in the Black African subjects as a result of cultural familiarity. This research achieves its general aim of studying one of the important issues in international marketing, by examining how consumer ethnocentrism studies can be beneficial in terms of using neuroscience tools such an ERP to supplement the traditional way of using scaled ranges of preferences and questionnaires.

## Ethics Statement

The experiment was conducted at Neuro-management lab, Zhejiang University. All subjects had normal or corrected to normal vision with no history of neurological or psychiatric abnormalities. This study was approved by the Neuromanagement Laboratory Ethics Committee at Zhejiang University. Written informed consent was obtained from all participants before the ERP experiment.

## Author Contributions

QM conceived the presented idea, verified the analytical methods and supervised the findings of this work. HA and LH carried out the experiment. HA wrote the manuscript with support from QM and LH. All authors discussed the results and contributed to the final manuscript.

## Conflict of Interest Statement

The authors declare that the research was conducted in the absence of any commercial or financial relationships that could be construed as a potential conflict of interest.

## Supplementary Material

TABLE S1 Comprise of seven datasets. (1) The age of the subject. (2) The rating score of S1. (3) The rating score of S2 (the race stimulus, S1 was eliminated). (4) The cultural sensitivity scale score. (5) The mean amplitude of P300 elicited by S1. (6) The mean amplitude of N2 elicited by S2. (7) The rating score of S2 (with S1 in the ERP experiment).
